# Neurenteric cysts: A neurosurgical case series and treatment perspectives

**DOI:** 10.37796/2211-8039.1652

**Published:** 2025-06-01

**Authors:** Defran Ercan, Walter Stummer, Maryam Khaleghi Ghadiri

**Affiliations:** Department of Neurosurgery, University Hospital Münster, 48149, Münster, Germany

**Keywords:** Neurenteric cyst, Brain lesion, Central nervous system, Spinal cord, Pain

## Abstract

Neurenteric cysts (NC) are rare lesions of endodermal origin lined by mucin-secreting cuboidal or columnar epithelium of an intestinal or respiratory type. They occur more frequently in the spinal cord than in the cranium. From the radiological view, NC may be confused with other lesions of the central nervous system, like arachnoid or epidermoid cysts. However, due to advances in neuroimaging, there is an increasing trend in preoperative diagnosis of NC. We are reporting three cases of NC, each exhibiting markedly distinct symptoms and postoperative courses. The first patient, a 45-year-old woman, had NC in the fourth ventricle. Although she underwent surgery, the lesion recurred 55 months later. The second patient, a 66-year-old woman, had NC in the left cerebellopontine angle. After partial removal, the remaining cyst maintained its size for 94 months. The third patient, a 55-year-old man, presented with NC close to the medullary cone. Despite tumor reduction, a hemorrhagic cyst developed 41 months later, which was accompanied by increased lumbosacral pain. A review of knowledge of current treatment strategies and challenges of NC is discussed. Surgical intervention is the primary therapeutic approach for patients with NC, particularly those experiencing symptoms. The primary challenge in treating NC is preventing cyst recurrence. The complete resection of NC is critical for minimizing the probability of cyst recurrence. Continuous lifelong follow-up is essential, as NC recurrences can occur even after decades.

## 1. Introduction

Neurenteric cysts (NC) are rare congenital malformations of endodermal origin. They were initially described as teratomatous cysts by Kubie and Fulton in 1928 [1]. The term “neurenteric cyst”, as it is currently used in the literature, was first introduced in 1954 by Holcomb and Matson [2]. These cysts are commonly found in the posterior mediastinum and account for about 0.01 % of all CNS tumors along the neuroaxis [3]. Due to their low incidence rate, NCs are mainly discussed in case reports. The treatment of these cysts has been a subject of controversy in the literature over the past few decades. So far, a standardized treatment concept is absent. This report has two aims: *(i)* to describe the clinical cases of three patients, and *(ii)* to explore current knowledge of NCs, particularly regarding available treatment options.

## 2. Methods

A retrospective study over a 12-year period was performed to include cases of intracranial NC operated at the Department of Neurosurgery of the University Hospital Münster from 2008 to 2020. A literature review was conducted to evaluate cases of NC. The search on PubMed/Medline, Mendeley, and Google Scholar included the following keywords: neurenteric cyst, neuroenteric cyst, endodermal cyst, enterogenous cyst, bronchogenic cyst, respiratory cyst, gastrocytoma cyst, neuroendodermal cyst, enterogenic cyst, teratomatous cyst, cranial, intracranial, spinal, and/or intraspinal. The abstracts of all identified investigations underwent assessment and only published manuscripts available online in English (in full text or with an abstract) were evaluated. Articles lacking adequate or sufficient data were excluded from evaluation.

## 3. Illustrative cases

### 3.1. Case 1

**Fig. 1:**
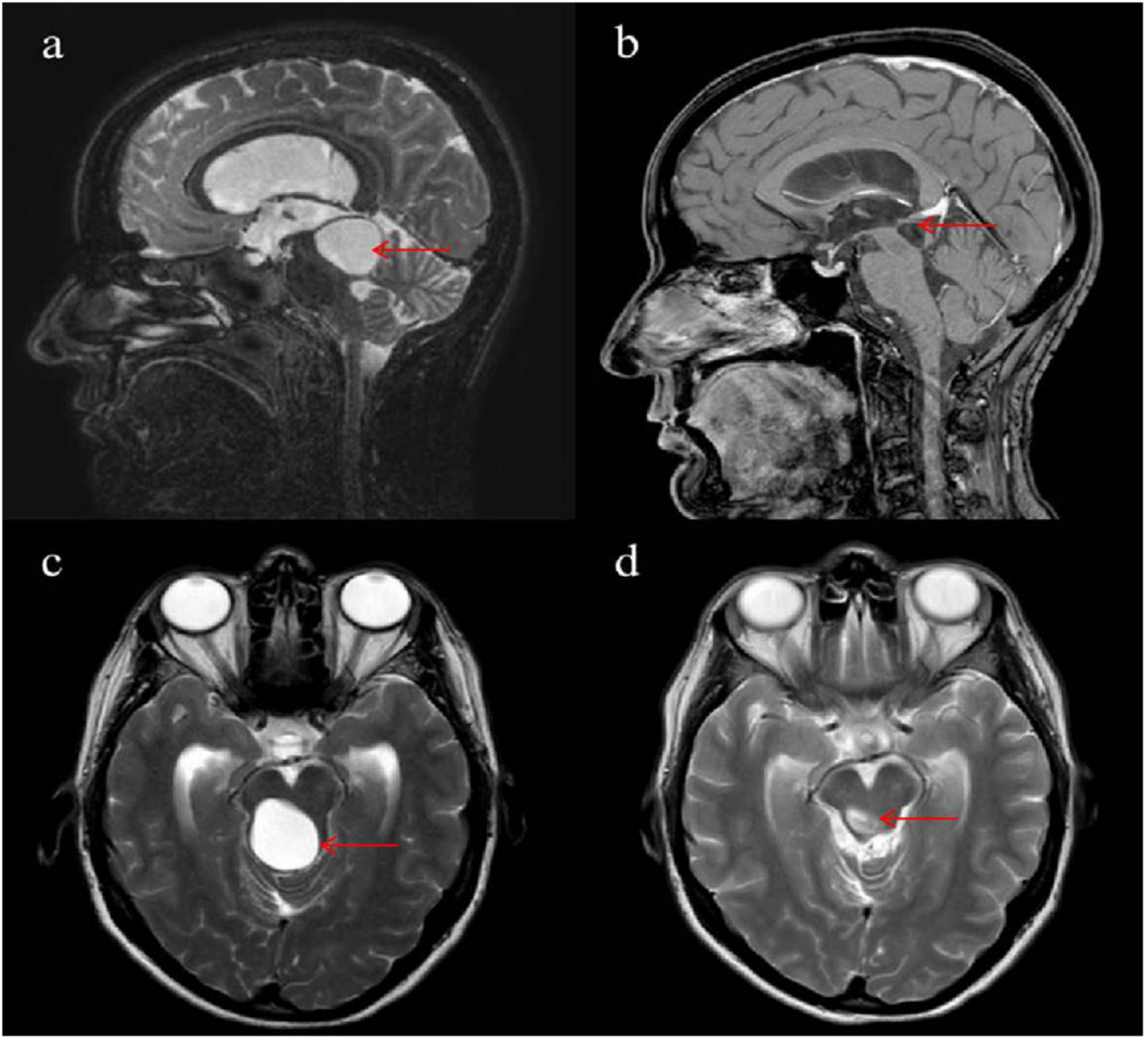
Brain MRI of case 1. Preoperative sagittal (a) and axial (c) T2-weighted MRI. Postoperative sagittal T1-weighted (b) and axial T2-weighted (d) MRI.

A 45-year-old woman diagnosed with prolactinoma presented with diplopia, abnormal fatigue, depression, and an unsteady gait. Apart from diplopia and walking towards the right during the gait test without any visual compensation, she exhibited no physical or neurological abnormalities. Brain magnetic resonance imaging (MRI) revealed a cyst in the fourth ventricle accompanied by obstructive hydrocephalus and the absence of a flow void in the aqueduct (Fig. 1a and c). The lesion was initially considered to be an arachnoid cyst, but it was observed to enlarge over a period of 14 years. The patient underwent surgery via a suboccipital approach. After the dura mater was opened, providing access to the fourth ventricle, the cyst was revealed. The cyst was opened and resected. The wall of the cyst was sent for histopathological assessments. There were no postoperative complications (Fig. 1b and d). Pathologic examination confirmed it as a NC. During the 55-month follow-up, a brain MRI revealed the recurrence of the cyst. The patient was in good condition but complained of paraesthesia on the tip of her tongue and her palate. She underwent a second surgery to remove the cyst. There were no intraoperative complications. Histopathological examination of the intraoperatively preserved tissue resulted in the diagnosis of reactively altered brain tissue. Parts of the previously diagnosed NC could not be identified (Fig. 1).

### 3.2. Case 2

**Fig. 2:**
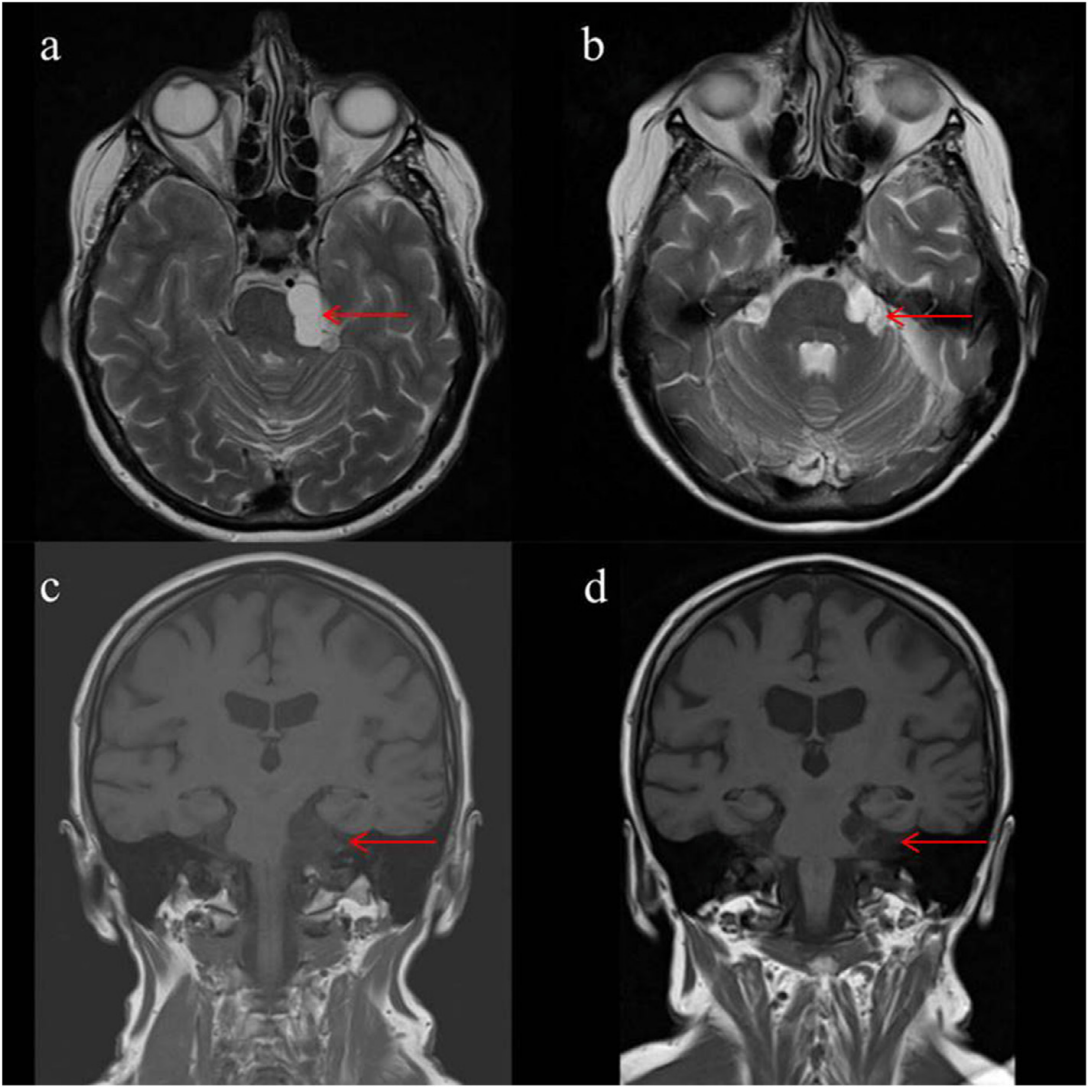
Brain MRI of case 2. Preoperative axial T2-weighted (a) and coronal T1-weighted (c) MRI. Postoperative axial T2-weighted (b) and coronal T1-weighted (d) MRI.

A 66-year-old woman presented with complaints of numbness on her left cheek, bilateral hypoacusis with greater severity on the left side, headaches, and vertigo over the past four weeks. One year earlier, imaging had revealed a cystic lesion in the left cerebellopontine angle. Neurological examination showed a left-sided hypesthesia in the area of the trigeminal nerve and hypoacusis. Brain MRI showed a multi-chambered space-occupying cystic lesion in the left cerebellopontine angle, measuring 1.5 × 1.8 × 2.5 cm (Fig. 2a and c). The patient underwent osteoclastic craniotomy for removal of the lesion using a suboccipital approach. No intraoperative complications occurred. Pathologic examination confirmed that the resected tissue was a NC. Postoperative MRI identified a residual cystic portion (Fig. 2b and d). During the initial three-month follow-up, the patient showed hypaesthesia in the area of the left trigeminal nerve, hypalgesia in the area of the maxillary nerve, facial paresis of House-Brackmann grade II, dysdiadochokinesia, unsteady gait, and headaches. Alongside the known residual cystic portion, a hygroma in the area of the left cerebellar hemisphere and microangiopathic changes were observed. During a follow-up period of 94 months, the extent of the residual cyst remained unchanged. After this period, she showed a slight improvement of the initial postoperative symptoms (Fig. 2).

### 3.3. Case 3

**Fig. 3:**
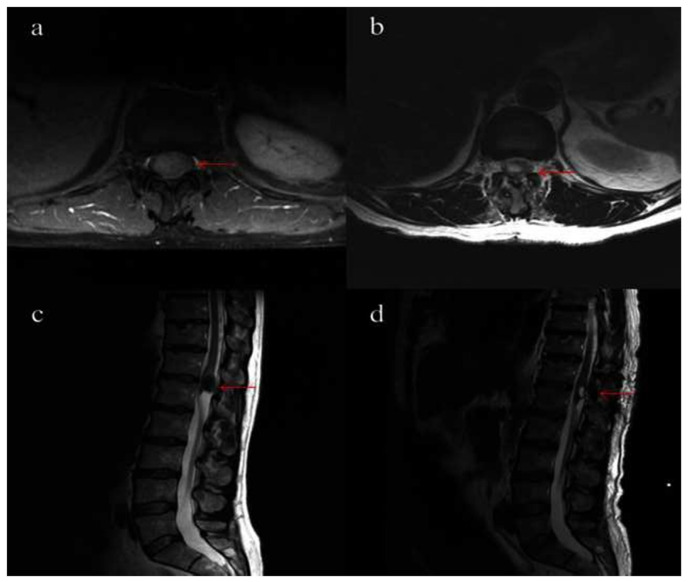
Spinal MRI of case 3. Preoperative axial T1-weighted (a) and sagittal T2-weighted (c) MRI. Postoperative axial (b) and sagittal (d) T2-weighted MRI.

A 55-year-old man presented to our hospital with worsening lumbago with sciatica and radicular pain along dermatomes L5 and S1 persisting for six months. In addition, he had a drop foot and a paraparesis with an ataxic gait. Physical examination showed lumbago with sciatica radiating out into the right buttock, unsteady gait, and loss of ankle jerk reflex. A spinal MRI revealed an intramedullary cystic lesion behind the first lumbar vertebra approximately 18 cm in length (Fig. 3a and c). Computer tomography showed the lesion in the area of the medullary cone with a dorsal orientation. The lesion was not calcified. A laminoplasty was performed. A lipomatous encapsulated process was observed and the cystic mass was opened. It contained necrotic, yellowish tumor tissue. Histopathological examination revealed the diagnosis of a NC. The postoperative recovery proceeded without complications. The postoperative MRI of the lumbar spine showed a considerable tumor reduction. A residual tumor was detected in the craniodorsal margin (Fig. 3b and d). The patient’s symptoms were relieved. After 41 months, the patient presented again because of an exacerbation of pain. The MRI showed a bled-in cyst in the region of the conus medullaris. The cyst remnants were removed during further surgery. Histopathologically, the diagnosis of a complex cyst was made, which had characteristics of an epidermoid cyst, but also showed features of a NC in other sections. After nine months, the patient complained of lumboischialgia again (Fig. 3).

## 4. Discussion

For symptomatic NC, prompt surgical intervention is considered crucial. Conservative therapies or even a wait-and-see approach are considered essential in patients with NC who display mild symptoms. However, surgical resection should also be considered for these patients. Permanent spinal cord compression or cyst rupture can lead to irreversible neurological deficits [4]. It has already been shown that only 23.0%–28.5% of NC are diagnosed preoperatively [5,6]. The surgical procedure for NC is not standardized. Lan et al. stated that the surgical approach should be individualized for each patient dependent on the location of NC [7]. In the literature, transoral, transclival, endoscopic endonasal, retrosigmoidal, suboccipital, and subtemporal approaches have been described for intracranial NC, depending on the exact position of the lesion [8,9]. Intraspinal NC are typically resected through a ventral, dorsal, or lateral approach to the spinal cord. However, due to the lower complication rate, a dorsal approach is preferred. Several surgical procedures for spinal NC are used, including laminotomy, laminectomy, hemilaminectomy, and laminoplasty [10]. Numerous authors propose that complete resection of NC, if possible, is advisable to prevent recurrence [11]. However, gross total resection (GTR) is not always possible due to the localization of the cyst. A GTR of intraspinal NC is more complicated by a ventral and/or intramedullary location, strong adhesion, and vertebral anomalies [12]. In contrast to those with an intramedullary location, GTR is easier to achieve in the case of intradural extramedullary localized NC, as the cyst wall can be more reliably differentiated from the spinal cord [13]. The differential diagnosis of NC should include consideration of other benign cystic lesions, such as the cystic dilatation of the ventriculus terminalis, particularly in the lumbar region. Accurate differentiation based on neuroimaging characteristics and anatomical location is crucial to providing proper management and avoiding unnecessary procedures [14,15]. The use of intraoperative neuromonitoring is recommended for intramedullary cysts [16]. Alternative approaches, such as simple aspiration, cyst fenestration, or marsupialization, can contribute to symptom improvement, however, many authors classify them as temporary measures [17]. Kim et al. only performed cyst fenestration. Despite this, no recurrence was found in the follow-up period of four years. They stated that the cyst fluid resembled cerebrospinal fluid and that this similarity could significantly contribute to the absence of recurrence even after cyst fenestration. Accordingly, cyst fenestration could be considered for NC with clear cyst contents in order to spare neurovascular structures [18]. In addition, the creation of a cystoarachnoid shunt is discussed in the literature [19]. Wang et al. used a technique to reduce the risk of aseptic or chemical meningitis. The cyst was first aspirated using a syringe and then the cyst wall was incised. The subarachnoid area, rostral and caudal to the lesion, should always be protected with cotton chips to prevent cyst contents from reaching this area. Furthermore, irrigation with a body-temperature saline solution is advised [5]. GTR was not achievable in our three patients. Significant improvement in symptoms postoperatively was observed in only one patient (case 1).

The data in the literature regarding the recurrence rate range from 0% to 50%. Intracranial NC develops recurrences more frequently, presumably due to the limitation of achieving GTR [18]. This could be also attributed to the greater extension of supratentorial cysts and thus a stronger adherence to neurovascular structures [20]. In addition to the already repeatedly verified thesis that STR of NC is the primary risk factor for the occurrence of a recurrence [21], there are already approaches that support a correlation between the frequency of recurrence and other factors. According to the studies by Chen and colleagues, there is a higher risk of recurrence in patients with an age of less than 30 years and a cyst size of more than 30 ml, especially within the first two years after surgical resection [20]. Weng et al. pointed that Wilkens and Odom type B or C NC had a greater likelihood of recurrence. They emphasized that these types of cysts have a higher rate of cyst fluid secretion compared to type A cysts [21]. Moreover, Wang et al. have suggested that the residual cyst wall is responsible for recurrence if it remains large enough to overlap again. Electro-coagulation of cyst wall remnants after STR may help to reduce the risk of recurrence [5]. Furthermore, Chen et al. have reported that most patients in their study developed recurrence in the first two years after initial treatment. In addition, another peak was observed after nine years. The reason for these two peaks is unclear [18]. Similar findings were reported by de Oliveira et al. and Can et al. [10,22] In keeping with our report, previous studies indicate that NC are recognized as slow-growing benign lesions with recurrence typically reported at approximately 7.8 years (ranging from 2 months to 32 years) [23]. Therefore, ongoing lifelong follow-up is suggested, as recurrence may occur decades after surgical intervention.

Currently, no evidence exists that radiotherapy reduces the risk of recurrence of a NC [24]. Adjuvant treatments are generally unnecessary. However, it should be noted that one patient with a recurrent intra-abdominal NC was successfully treated with Picibanil (OK-432), an immunotherapeutic substance [25]. Cerebrospinal fluid testing may be helpful, as elevated levels of CA 19-9 may signal recurrence [26]. A recurrent NC is technically difficult to resect due to the inflammatory response caused by epithelial secretion [27]. Because of this, prompt reoperation is recommended if a residual cyst shows expansion or presents with neurological deficits [28]. Furthermore, the utilization of neuronavigation technology and adherence to microsurgical principles are advised during surgery for NC to accomplish complete lesion resection [29–31]. Moreover, it is crucial to note that neuroimaging might reveal similarities between NC and other cystic lesions, such as arachnoid cysts or epidermoid cysts, possibly resulting in misdiagnosis [32]. In our cases, recurrence occurred in the first patient after 55 months and in the third patient 41 months after the first surgery. The NC in the cerebellopontine angle did not recur in a follow-up period of 94 months despite performing STR.

## 5. Conclusion

Surgical resection of NC in our cases markedly alleviated the initial complaints, although certain symptoms persisted even after several years postoperation. This indicates that surgical resection ofNC is an appropriate way to treat patients with NC. The choice of a patient-specific surgical approach should be determined based on the localizations, with the aim of GTR. The recurrence rate is significantly associated with subtotal resection. Adjuvant treatments in addition to surgical resection seem to be unnecessary. This report is limited by its retrospective nature and the small number of featured cases. However, we believe that these illustrative cases contribute to the existing body of knowledge of NC.

## Data Availability

Data are available upon request.
